# Optineurin Is Required for CYLD-Dependent Inhibition of TNFα-Induced NF-κB Activation

**DOI:** 10.1371/journal.pone.0017477

**Published:** 2011-03-07

**Authors:** Ananthamurthy Nagabhushana, Megha Bansal, Ghanshyam Swarup

**Affiliations:** Centre for Cellular and Molecular Biology, Council of Scientific and Industrial Research, Hyderabad, India; Johns Hopkins School of Medicine, United States of America

## Abstract

The nuclear factor kappa B (NF-κB) regulates genes that function in diverse cellular processes like inflammation, immunity and cell survival. The activation of NF-κB is tightly controlled and the deubiquitinase CYLD has emerged as a key negative regulator of NF-κB signalling. Optineurin, mutated in certain glaucomas and amyotrophic lateral sclerosis, is also a negative regulator of NF-κB activation. It competes with NEMO (NF-κB essential modulator) for binding to ubiquitinated RIP (receptor interacting protein) to prevent NF-κB activation. Recently we identified CYLD as optineurin-interacting protein. Here we have analysed the functional significance of interaction of optineurin with CYLD. Our results show that a glaucoma-associated mutant of optineurin, H486R, is altered in its interaction with CYLD. Unlike wild-type optineurin, the H486R mutant did not inhibit tumour necrosis factor α (TNFα)-induced NF-κB activation. CYLD mediated inhibition of TNFα-induced NF-κB activation was abrogated by expression of the H486R mutant. Upon knockdown of optineurin, CYLD was unable to inhibit TNFα-induced NF-κB activation and showed drastically reduced interaction with ubiquitinated RIP. The level of ubiquitinated RIP was increased in optineurin knockdown cells. Deubiquitination of RIP by over-expressed CYLD was abrogated in optineurin knockdown cells. These results suggest that optineurin regulates NF-κB activation by mediating interaction of CYLD with ubiquitinated RIP thus facilitating deubiquitination of RIP.

## Introduction

Nuclear factor-κB (NF-κB) plays a key role in the expression of many genes involved in regulating immune response, apoptosis, cell cycle and its deregulation is involved in the pathogenesis of many diseases [1], [2]. In unstimulated cells, NF-κB is sequestered in the cytoplasm through its association with the inhibitory IκB proteins. During activation of NF-κB by the cytokine tumor necrosis factor (TNF)α, signalling intermediates like TRADD (TNF receptor associated death domain), TRAF2 (TNF receptor associated factor) and RIP (receptor interacting protein) are recruited to the TNF receptor (TNFR1). This results in the activation of IκB kinase complex (IKK) consisting of the catalytic IKKα and β subunits and the regulatory subunit IKK-γ/NEMO (NF-κB essential modulator). IKK activation involves conjugation of Lys63-linked polyubiquitin chains to NEMO and its upstream regulators like RIP [3]. RIP has emerged as a central adaptor in the pathways leading to IKK and NF-κB activation and also cell death. Following TNFα stimulation RIP is recruited to TNFR1 signalling complex and is rapidly ubiquitinated with Lys63-linked polyubiquitin chains. NEMO binds to polyubiquitinated RIP through its ubiquitin binding domain (UBD) resulting in the activation of catalytic subunits of IKK. The recognition and association of ubiquitinated RIP with NEMO is essential for IKK activation [4]–[6]. The activated catalytic subunits of IKK then phosphorylate IκB triggering its ubiquitination and degradation leading to nuclear translocation and activation of NF-κB.

Given its role in diverse cellular processes, the activation of NF-κB is governed by several positive and negative regulators. With the increasing role of ubiquitination, deubiquitinases like CYLD and A20 have emerged as key negative regulators of NF-κB activation [3], [7]–[12]. CYLD was originally identified as a tumor suppressor gene mutated in familial cylindromas [13]. It is the first deubiquitinase shown to inhibit IKK activation [7]–[9]. CYLD specifically catalyses cleavage of Lys63-linked polyubiquitin chains from its target proteins like RIP, NEMO and TRAFs to prevent NF-κB activation [7]–[10], [14], [15]. Though CYLD targets multiple NF-κB signalling molecules, the mechanism by which CYLD recognises its substrate RIP to regulate NF-κB activation is not completely understood.

Optineurin was recently identified as a negative regulator of NF-κB signalling whose expression is governed by NF-κB [16]–[18]. It is a multifunctional protein involved in membrane trafficking, signal transduction, anti-viral responses and gene expression [16], [18]–[26]. The C-terminal region of optineurin has a novel bipartite UBD which shows homology with NEMO and ABIN1 [16], [27]. This UBD of Optineurin, like NEMO, preferentially binds to Lys63-linked ubiquitin chains and does not show significant binding to Lys48-linked polyubiquitin chains [16]. It was suggested that optineurin binds to polyubiquitinated RIP through its UBD to prevent association of NEMO with RIP, thus inhibiting NF-κB activation [16]. Optineurin was identified as a gene mutated in certain glaucomas, a group of neurodegenerative eye diseases that cause blindness, and recently in familial amyotrophic lateral sclerosis [28], [29]. However, the nature of functional defects caused by mutations in optineurin is beginning to be understood only now [21], [29]–[31].

Recently we have identified CYLD as an interacting protein of optineurin in a yeast-two hybrid screen. This was reported briefly in a review without showing any data [32]. However the functional significance of this interaction is not known. Since optineurin interacts with CYLD, the role of optineurin in the regulation of NF-κB signalling is likely to be complex. Here we have analysed the role of optineurin-CYLD interaction in the regulation of TNFα-induced NF-κB activation. A glaucoma-associated mutant of optineurin (H486R) shows reduced binding to CYLD. This mutant, unlike wild type optineurin, does not inhibit TNFα-induced NF-κB activation. Optineurin is essential both for inhibition of TNFα-induced NF-κB activation by CYLD and its association with RIP. In addition we show that optineurin is required for CYLD mediated deubiquitnation of RIP. Our results thus show that the interaction of optineurin with CYLD is important for the regulation of TNFα-induced NF-κB activity.

## Results

### C-terminal domain of Optineurin interacts with CYLD

**Figure 1:**
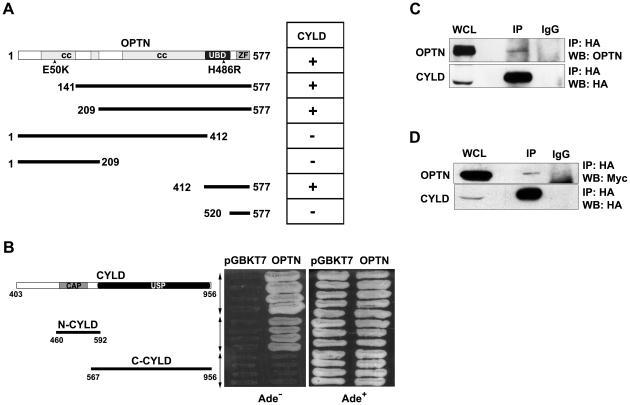
C-terminal region of optineurin interacts with CYLD. Interaction of optineurin and its deletion constructs with CYLD was analysed using yeast two-hybrid method. Interaction is denoted by ‘+’. CC, coiled coil; UBD, ubiquitin binding domain; ZF, zinc finger. Yeast strain PJ69-4A was co-transformed with optineurin and CYLD or its deletion constructs. Transformants were grown on selection media lacking Ade to assay interaction. Growth on Ade^−^ plate indicates interaction. CAP, CAP-Gly domain; USP, ubiquitin specific protease. HeLa cells were transfected with HA-CYLD and CYLD was immunoprecipitated using HA tag antbody. Immunoprecipitates were analyzed by western blotting with HA and optineurin antibodies. OPTN, optineurin; WCL, whole cell lysate. HeLa cells were co-transfected with Myc-OPTN and HA-CYLD. CYLD was immunoprecipitated with HA antibody and immunoprecipitates were analyzed by western blotting with HA and Myc antibodies.

CYLD was identified as optineurin-interacting protein by yeast two-hybrid screening using full length optineurin as bait [32]. The cDNA clone obtained codes for amino acids 403–956 of CYLD. Deletion analysis showed that the C-terminal domain of optineurin (412–577 amino acid) was involved in binding to CYLD (Figure 1A). This C-terminal region of optineurin encompasses the UBD (424–509 amino acids) through which it interacts with ubiquitinated RIP. Similarly deletion of various domains of CYLD showed that amino acids 460 to 592 of CYLD were sufficient for interaction with optineurin (Figure 1B). The catalytic domain of CYLD was dispensable for the interaction with optineurin (Figure 1B). Interestingly, NEMO with which optineurin shows ∼53% sequence identity was also shown to interact with similar region (aa 470–684) of CYLD in yeast-two hybrid assay [9]. To examine the interaction of CYLD with optineurin in mammalian cells, co-immunoprecipitation assays were performed. HeLa cells were transfected with HA-tagged CYLD expression plasmid and after 36 hours cell lysates were subjected to immunoprecipitation with HA antibody. Endogenous optineurin was detected in the immunoprecipitate with HA antibody but not in the immunoprecipitate with control antibody (Figure 1C). Similarly Myc-optineurin could co-immunoprecipitate with over-expressed CYLD (Figure 1D). These results suggest that endogenous as well as overexpressed optineurin interacts with CYLD in mammalian cells.

### The H486R mutant of optineurin shows altered interaction with CYLD

**Figure 2:**
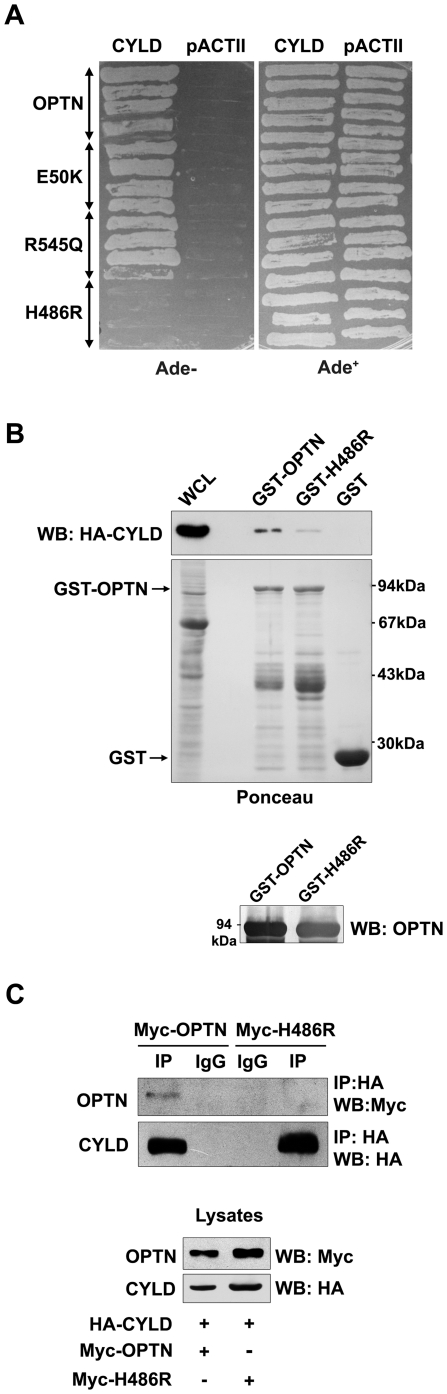
H486R optineurin is defective in interaction with CYLD. Yeast strain PJ69-4A was co-transformed with wild-type optineurin or its mutants and CYLD. Transformants were grown on selection media lacking adenine to assay interaction. GST-optineurin, GST-H486R or GST alone bound to glutathione agarose beads were incubated with lysates of HeLa cells transfected with HA-CYLD. The bound proteins were eluted and immunoblotted with anti-HA antibodies. Western blot was done with optineurin antibody (lower panel) to confirm the identity of the GST fusion proteins. HeLa cells were co-transfected with HA-CYLD and Myc-OPTN or Myc-H486R. Lysates were immunoprecipitated with anti-HA antibody and subjected to western blotting. WCL, whole cell lysate.

We tested the hypothesis that some of the glaucoma-associated mutants of optineurin may be altered in their interaction with CYLD. Wild-type optineurin or its glaucoma-associated mutants (E50K, H486R and R545Q) were co-transformed with CYLD in yeast. One of the glaucoma-associated mutants, H486R, failed to interact with CYLD (Figure 2A). *In vitro* binding assays using GST-optineurin and GST-H486R with cell lysates expressing HA-CYLD were carried out. As compared with wild type optineurin, the H486R mutant showed 5-fold less binding with HA-CYLD (Figure 2B). These observations were further validated by co-imunoprecipitation. The H486R mutant showed less interaction with HA-CYLD than wild type optineurin (Figure 2C). Optineurin is present in the cell as a high molecular weight homo-hexameric complex perhaps in association with other proteins which may influence its interaction with CYLD [33]. Overall our binding experiments suggest that the H486R mutant is altered in its interaction with CYLD.

### The H486R mutant does not inhibit TNFα-induced NF-κB activation

**Figure 3:**
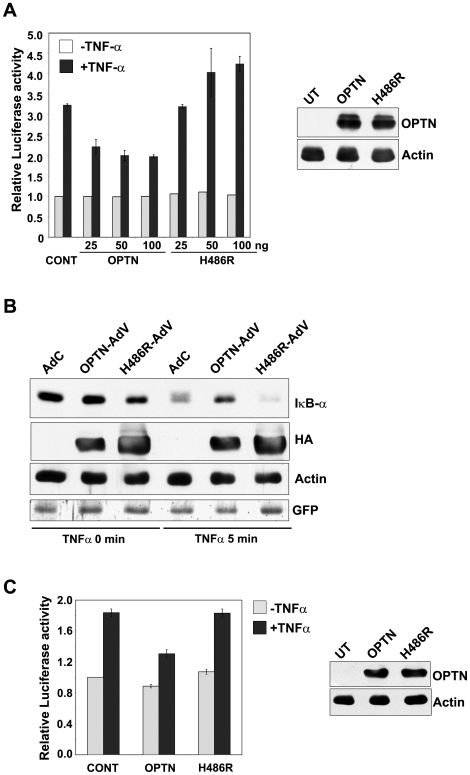
H486R mutant does not inhibit TNFα- induced NF-κB activation. HeLa cells were transfected with optineurin or its H486R mutant (25, 50 or 100 ng) along with NF-κB reporter plasmid and, after 22 h, treated with TNFα for 4 h. Luciferase activities relative to untreated control are shown (n = 4). Western blot shows expression of optineurin and its H486R mutant using HA tag antibody. HeLa cells were infected with adenoviruses for expressing HA tagged optineurin (Optn-AdV) or H486R (H486R-AdV) mutant or control virus (AdC). After 36 hours of infection, the cells were treated with TNFα for 5 min or left untreated. Cell lysates were then prepared for western blotting with antibodies for IκBα, HA tag and actin (loading control). GFP expression was used to monitor infection by adenoviruses. RGC-5 cells were transfected with optineurin or its H486R mutant (100 ng) along with NF-κB reporter plasmid and, after 22 h of transfection, treated with TNFα for 3 h. Luciferase activities relative to untreated control are shown (n = 4). Western blot shows expression of optineurin and its H486R mutant using HA tag antibody.

As H486R mutant was altered in its interaction with CYLD, we studied the effect of optineurin and its H486R mutant on TNFα-induced NF-κB activation. HeLa cells were transfected with an NF-κB luciferase reporter construct along with or without optineurin expression plasmid or its mutant. After 22 hours of transfections, the cells were treated with TNFα for 4 hours and the lysates were assayed for luciferase activity. Neither wild-type optineurin nor H486R mutant inhibited basal NF-κB activity. In response to TNFα, NF-κB reporter activity was increased to 3.2 fold. TNFα-induced NF-κB activation was partially inhibited by wild-type optineurin (Figure 3A). This inhibition by optineurin was statistically significant at all the three concentrations of optineurin tested (P<0.05, n = 4). However, the H486R mutant failed to inhibit TNFα-induced NF-κB activation (Figure 3A). The expression of optineurin and its mutant was confirmed by western blotting (Figure 3A). These observations suggest that the H486R mutant has lost the ability to inhibit NF-κB activation induced by TNFα.

Stimulation of cells with TNFα, results in rapid phosphorylation of IκB-α by the IKK complex. The phosphorylation of IκB-α acts as a trigger for its degradation by proteasome, leading to nuclear translocation of NF-κB subunits where they regulate the expression of target genes [1]. Since overexpression of H486R mutant did not inhibit TNFα-induced NF-κB activation, we investigated the effect of optineurin and its H486R mutant on IκB-α degradation. A significant amount of IκB-α was degraded by 5 min of TNFα-treatment in control cells. TNFα-induced IκB-α degradation was partially inhibited upon overexpression of optineurin (Figure 3B). However, expression of H486R optineurin did not inhibit TNFα-induced IκB-α degradation (Figure 3B). GFP expressed by the adenoviruses was used to monitor infection by adenoviruses (Figure 3B). These results are consistent with the suggestion that the H486R mutant does not inhibit TNFα-induced NF-κB activation.

We also examined the effect of optineurin on TNFα-induced NF-κB activation in a retinal ganglion cell line RGC-5, a neuronal cell line relevant for glaucoma. Expression of wild type optineurin in RGC-5 cell resulted in significant inhibition (P<0.05, n = 4) of TNFα-induced NF-κB activity whereas the H486R mutant showed no significant inhibition (Figure 3C).

### Effect of H486R optineurin on CYLD-dependent inhibition of TNFα-induced NF-κB activity

**Figure 4:**
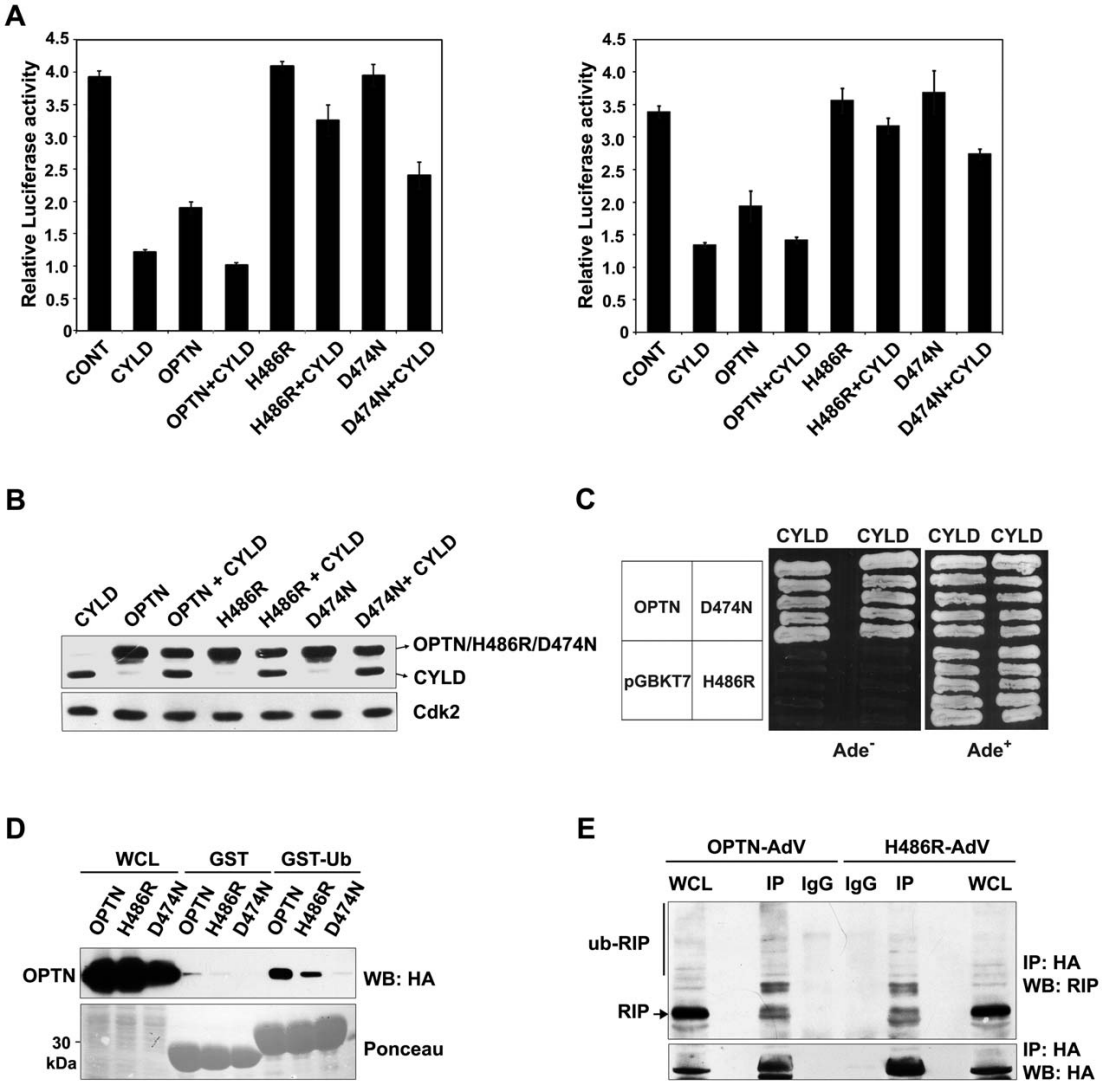
Effect of H486R optineurin on CYLD-dependent inhibition of TNFα-induced NF-κB activity. HeLa cells were transfected with optineurin or its mutants (100 ng) along with or without CYLD (100 ng, left panel and 50 ng, right panel). After 22 h of transfection, the cells were treated with TNFα for 4 h. Luciferase activities relative to untreated control are shown (n = 4). Western blot showing the expression of optineurin and its mutants along with CYLD using HA tag antibody. Yeast strain PJ694A was co-transformed with optineurin or its H486R and D474N mutants and CYLD. Transformants were grown on selection media lacking Ade to assay interaction. Growth on Ade^−^ plate indicates interaction. GST-ubiquitin or GST alone bound to glutathione agarose beads were incubated with lysates of HEK293T cells transfected with wild type optineurin or its mutants. The bound proteins were eluted and immunoblotted with anti-HA antibodies. WCL, whole cell lysates. HeLa cells were infected with adenoviruses expressing HA-tagged wild-type or H486R mutant optineurin. After 30 hrs of infection, the cells were treated with TNFα for 5 min and immunoprecipitations were carried out with HA antibody and analyzed by Western blotting with RIP and HA antibodies.

We next analysed the effect of expression of the H486R mutant and wild type optineurin on CYLD mediated inhibition of TNFα-induced NF-κB activation. Overexpression of CYLD resulted in strong (80–100%) inhibition of NF-κB activation by TNFα (Figure 4A). In presence of the H486R mutant, overexpressed CYLD showed only 10–20% inhibition of TNFα-induced NF-κB activity whereas in presence of wild type optineurin CYLD showed 80–100% inhibition (Figure 4A). This was not due to lower expression of CYLD in presence of H486R mutant optineurin as shown by western blot of the same samples (Figure 4B). These results show that overexpressed H486R optineurin prevents CYLD- mediated inhibition of TNFα-induced NF-κB activity.

Optineurin negatively regulates TNFα-induced NF-κB activation by competing with NEMO for binding of ubiquitinated RIP through its UBD. Inactivation of UBD of optineurin by a point mutation (D474N) results in loss of inhibition of NF-κB activation [16]. The D474N mutant, however, was not defective in its interaction with CYLD as revealed by yeast-two hybrid assays (Figure 4C). Since the H486R mutation is also present in the UBD, it is possible that this mutant may be defective in ubiquitin binding. Compared to wild-type optineurin, the H486R mutant showed reduced binding to GST-ubiquitin whereas the D474N mutant did not show any binding (Figure 4D). The ability of H486R mutant optineurin to bind ubiquitinated RIP was tested by co-immunoprecipitation. As compared to wild-type optineurin, the H486R mutant showed some reduction in binding to ubiquitinated RIP in TNFα- treated cells (Figure 4E). These results suggest that the loss of interaction with CYLD is the major reason for the reduced inhibition of TNFα-induced NF-κB activation by the H486R mutant. However, the reduced binding to ubiquitin may also contribute towards its effect on CYLD-dependent inhibition of NF-κB activation. In presence of the D474N mutant, overexpressed CYLD showed partial inhibition of TNFα-induced NF-κB activation (Figure 4A). Thus as compared to the H486R mutant, the D474N mutant is less effective in preventing CYLD mediated inhibition of NF-κB activation. This may be due to the fact that while H486R is defective in binding to CYLD and also shows reduced binding to ubiquitin, the D474N is defective in only ubiquitin binding. Since D474N mutant does not bind ubiquitinated RIP [16] this suggests that the interaction of UBD of optineurin with ubiquitinated proteins (possibly RIP) also plays an essential role in CYLD mediated inhibition of TNFα- induced NF-κB activation.

### Optineurin is essential for CYLD mediated inhibition of NF-κB activation

**Figure 5:**
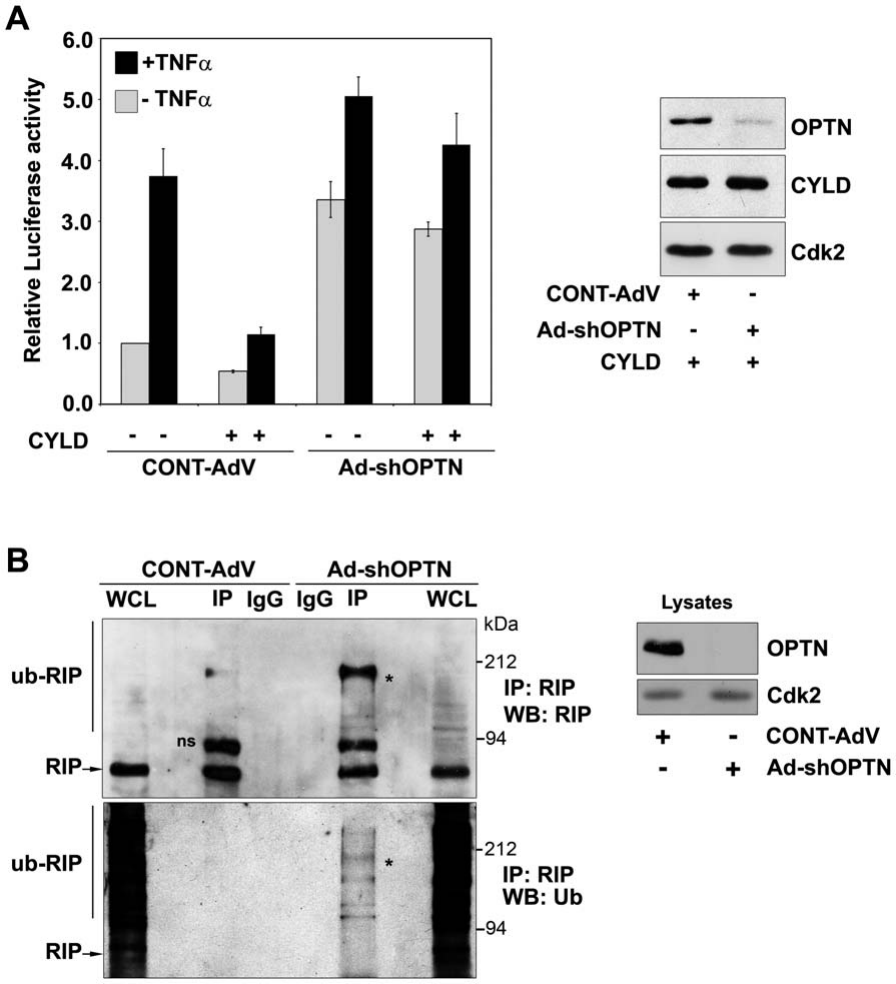
Optineurin is required for CYLD mediated inhibition of NF-κB activation. HeLa cells were infected with adenoviruses expressing shRNA against optineurin or with control adenoviruses. After 48 hrs of infection cells were transfected with NF-κB-Luc with or without CYLD (100 ng). The cells were treated with TNFα for 4 h after 22 h of transfection. Luciferase activities relative to untreated control are shown (n = 4). Western blot shows the expression of HA-CYLD in control and optineurin knockdown cells. RIP ubiquitination is enhanced in optineurin knockdown cells. HeLa cells were infected with Ad-shOPTN or with control (Cont-AdV) adenoviruses. After 72 h of infection cell lysates were subjected to immunoprecipitation with RIP antibody or control antibody. The immunoprecipitates were analyzed by western blotting. ns, non-specific. * indicates ubiquitinated RIP.

We next examined the requirement of optineurin in CYLD mediated abrogation of TNFα-induced NF-κB activation. HeLa cells were infected with adenoviruses expressing shRNA against optineurin or with control adenoviruses. After 48 h of infection, these cells were transfected with CYLD along with NF-κB luciferase reporter and treated with TNFα for 4 h after 22 h of transfection. As reported earlier, optineurin knockdown enhanced both basal as well as TNFα-induced NF-κB activity [16], [18]. Overexpression of CYLD resulted in strong inhibition of both basal and TNFα-induced NF-κB activity in control cells (Figure 5A). However, upon knockdown of optineurin, overexpression of CYLD resulted in only a marginal inhibition of basal and TNFα-induced NF-κB activity (Figure 5A). These results show that optineurin is essential for CYLD mediated inhibition of basal and TNFα- induced NF-κB activity.

The mechanism by which knockdown of optineurin causes increase in basal NF-κB activity is not clear. The finding that the increased NF-κB activity is not inhibited by over-expressed CYLD prompted us to investigate whether loss of optineurin affects levels of ubiquitinated RIP. RIP was immunoprecipitated from optineurin knockdown and control cells in presence of N-ethylmaleimide (to prevent deubiquitination) and the immunoprecipitates were analysed by western blot using RIP and ubiquitin antibody. The level of ubiquitinated RIP was increased in optineurin knockdown cells (Figure 5B). These results suggest that in optineurin knockdown cells enhanced basal NF-κB activity is likely to be due to increased level of ubiquitinated RIP.

### Optineurin is required for CYLD-dependent deubiquitnation of RIP

**Figure 6:**
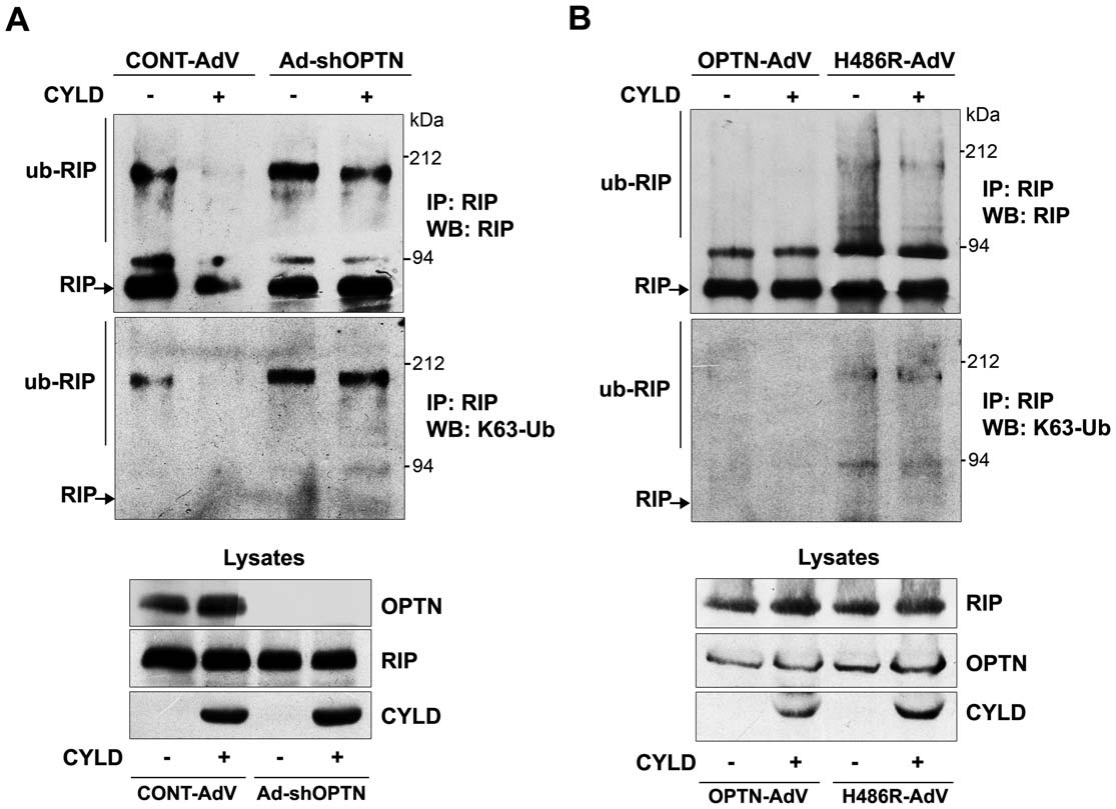
Optineurin is required for deubiquitination of RIP by CYLD. HeLa cells were infected with adenoviruses expressing shRNA against optineurin or with control adenoviruses. After 48 hrs of infection, cells were either left untransfected or transfected with HA- CYLD, treated with TNFα for 10 min after 30 h of transfection and subjected to immunoprecipitation with RIP or control antibodies. Immunoprecipitates were then subjected to western blotting with RIP antibody. The blot was then reprobed with Lys63-linked polyubiquitin (K63-Ub) antibody. HeLa cells were infected with adenoviruses expressing wild-type optineurin or H486R mutant. After 4 h of infection, cells were either left untransfected or transfected with HA- CYLD, treated with TNFα for 10 min after 24 h of transfection and subjected to immunoprecipitation with RIP or control antibodies. Immunoprecipitates were then subjected to western blotting with RIP antibody. The blot was then reprobed with Lys63-linked polyubiquitin (K63-Ub) antibody.

Since optineurin knockdown cells accumulate higher levels of ubiquitinated RIP and CYLD fails to abolish NF-κB activation, it is plausible that optineurin is required for deubiquitination of RIP by CYLD. To test this assumption, deubiquitination of RIP by overexpressed CYLD was examined in control and optineurin knockdown cells. HeLa cells were infected with adenoviruses expressing shRNA against optineurin or with control adenoviruses. After 48 h of infection, cells were transfected with CYLD expression plasmid or left untransfected and treated with TNFα for 10 min after 30 h of transfection. RIP was immunoprecipitated and the immunoprecipitates were analysed by western blotting with RIP antibody followed by reprobing of the blot with Lys63-specific polyubiquitin antibody. In control cells, only a small fraction of RIP was found polyubiquitinated upon over-expression of CYLD indicating that RIP was efficiently deubiquitinated by CYLD (Figure 6A). However, in optineurin depleted cells level of polyubiquitinated RIP was higher and overexpression of CYLD resulted in only a small decrease in the level of ubiquitinated RIP (Figure 6A). This was not due to lower level of CYLD expression in optineurin knockdown cells. These results suggest that CYLD fails to deubiquitinate RIP in the absence of optineurin.

As CYLD could not inhibit NF-κB activation in H486R expressing cells, we next examined whether CYLD can deubiquitinate RIP in presence of H486R mutant. HeLa cells were infected with adenoviruses expressing HA-tagged wildtype- or H486R mutant optineurin and transfected with CYLD expression plasmid or left untransfected. The cells were treated with TNFα for 10 min after 24 h of transfection and RIP was immunoprecipitated. The immunoprecipitates were then analysed by western blotting with RIP antibody followed by reprobing of the blot with Lys63-specific polyubiquitin antibody. In cells expressing wild-type optineurin, only a small amount of RIP was found polyubiquitinated. Upon co-expression of CYLD in these cells, we were not able to detect ubiquitinated RIP (Figure 6B). In cells expressing the H486R mutant, level of polyubiquitinated RIP was higher compared to wild-type optineurin expressing cells and co-expression of CYLD resulted in very small decrease in the level of ubiquitinated RIP, as detected by Lys63-specific polyubiquitin antibody (Figure 6B). This was not due to lower expression of either the H486R mutant or CYLD (Figure 6B). These observations suggest that the H486R mutant prevents CYLD mediated deubiquitination of RIP.

### Optineurin is essential for interaction of CYLD with ubiquitinated RIP

**Figure 7:**
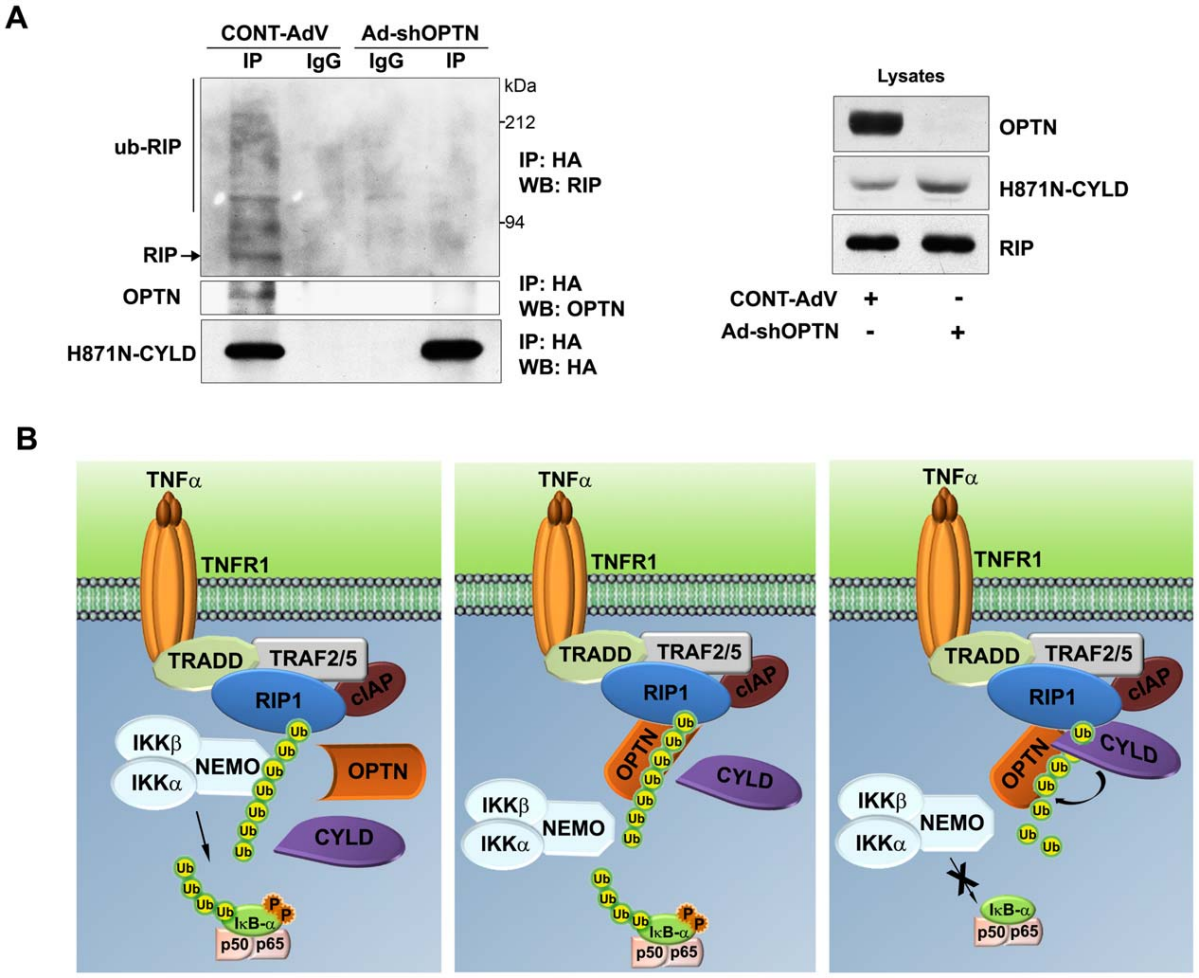
Optineurin is required for interaction of CYLD with RIP. HeLa cells were infected with adenoviruses expressing shRNA against optineurin or with control adenoviruses. After 48 h of infection, cells were transfected with HA-tagged mutant (H871N) CYLD, treated with TNFα for 5 min after 30 h of transfection and subjected to immunoprecipitation with HA or control antibodies. Immunoprecipitates were then subjected to western blotting. A schematic representing the regulation of TNFα-induced NF-κB signalling by optineurin. Binding of TNFα to its receptor leads to assembly of a multimolecular complex on TNF receptor in which ubiquitination of RIP takes place. Then NEMO is recruited to ubiquitinated RIP. This leads to activation of IKK (left panel). Optineurin binds to ubiquitinated RIP possibly by displacing NEMO (middle panel) as suggested by Zhu et al [16] and then recruits CYLD to the molecular complex thus facilitating deubiquitination of RIP by CYLD (right panel). In the absence of optineurin, CYLD is not recruited to ubiquitinated RIP resulting in accumulation of ubiquitinated RIP.

Since CYLD failed to deubiquitinate RIP in the absence of optineurin, it is likely that optineurin acts as an adaptor to facilitate interaction of CYLD with RIP. To test this possibility, endogenous optineurin in HeLa cells was knocked down using shRNA and then these cells were transfected with a catalytically inactive mutant (H871N) of CYLD. After 30 hrs of transfection, these cells were treated with TNFα for 5 min and CYLD was immunoprecipitated. Ubiquitinated RIP and CYLD could co-immunoprecipitate along with endogenous optineurin in control cells indicating that CYLD, RIP and optineurin exist in a single complex. Upon knockdown of optineurin the ability of CYLD to associate with ubiquitinated RIP was drastically reduced (Figure 7A). The catalytically inactive mutant of CYLD was used for this experiment because we got very little or no ubiquitinated RIP in the pulldown with wild type CYLD. These results show that optineurin is essential for interaction of CYLD with ubiquitinated RIP. In conclusion, our observations strongly suggest that optineurin mediates targeting of CYLD to its substrate (i.e ubiquitinated RIP) which facilitates its deubiquitination.

## Discussion

Ubiquitin binding proteins, with their diverse range of UBDs, have emerged as key regulators of NF-κB signalling. Optineurin was shown to inhibit TNFα-induced NF-κB activation by competing with NEMO for the binding of ubiquitinated RIP [16]. However, our results suggest that the regulation of NF-κB activation by optineurin is more complex and binding of ubiquitinated RIP to UBD of optineurin is only one of the steps in this complex regulation. Although ubiquitinated RIP is known to be a substrate of CYLD, the mechanism by which CYLD is recruited to RIP to deubiquitinate it, is not clear [14]. The results presented in this manuscript suggest that optineurin mediates interaction of deubiquitinase CYLD with polyubiquitinated RIP and this interaction is essential for deubiquitination of RIP by CYLD. Thus an important function of optineurin in the regulation of NF-κB signalling is to act as an adaptor protein bringing CYLD and its substrate RIP together to facilitate deubiquitination of ubiquitinated RIP by CYLD.

CYLD targets multiple players of the NF-κB signalling pathway. How CYLD recognises and is targeted to a specific substrate like RIP is not well understood. CYLD interacts directly with some of its substrates like TRAF2 and NEMO [7]–[9]. Emerging evidence suggests that CYLD might indirectly associate with some of its substrates through intermediary adaptor proteins. It has been shown that p62/sequestosome1 binds to TRAF6 through its UBD and recruits CYLD to TRAF6 to regulate its ubiquitination [34], [35]. An adaptor protein should be able to specifically bind to substrates polyubiquitinated with Lys63-linked ubiquitin chains since CYLD specifically deconjugates Lys63-linked polyubiqitin chains. In addition, such an adaptor protein should also interact with CYLD. Since optineurin interacts with Lys63-linked polyubiquitin chains (as in ubiquitinated RIP) through its UBD and also with CYLD, it is ideally suited to act as an adaptor for CYLD to recognise its substrates. The ABIN proteins with their homologous UBD similarly act as adaptors for the recruitment of A20 to its targets like NEMO [36]. TNFα stimulus triggers assembly of Lys63-linked polyubiquitin chains on RIP which initially binds to NEMO to activate IKK complex. Optineurin binds to polyubiquitinated RIP through its UBD and recruits CYLD; this may prevent association of NEMO with RIP (Figure 7B). Close proximity of binding sites of both CYLD and RIP on optineurin provides support for the adaptor function of optineurin in facilitating interaction of RIP and CYLD. These assumptions are strengthened by our observations which show that both inhibition of NF-κB activation by CYLD and association of CYLD with RIP are dependent on optineurin. Consistent with its role as an adaptor facilitating the association of a deubiquitinase with its substrate, depletion of optineurin impaired deubiquitination of RIP by CYLD. Whether optineurin plays a role in recruiting CYLD to its other targets is yet to be investigated.

Knockdown of endogenous optineurin increases basal level of NF-κB activity in unstimulated cells [16], [18] but the mechanism of this regulation was not clear. CYLD deficiency results in the accumulation of its ubiquitinated targets like RIP due to constitutive ubiquitination [10], [14]. Hence it has been suggested that CYLD is a constitutively active deubiquitinase that prevents spontaneous ubiquitination of its targets like RIP, thus maintaining low basal NF-κB activity. Our results show that optineurin depleted cells accumulate higher levels of ubiquitinated RIP. This elevated level of RIP ubiquitination is likely to be due to inability of CYLD to associate with and deubiquitinate RIP in the absence of optineurin leading to enhanced NF-κB activation (Figure 7B). The elevated level of ubiquitinated RIP in optineurin depleted cells differs/is not consistent with the model of Zhu et al [16]. However, these observations are consistent with our proposed model of optineurin as an adaptor protein, as depicted in Figure 7B. The main feature of this model is that optineurin mediates binding of CYLD to ubiquitinated RIP. This model incorporates the findings of Zhu et al [16] that optineurin binds to ubiquitinated RIP by displacing NEMO. In fact our work/model can also be viewed as an extension of the work of Zhu et al [16].

Some of the data presented in this study are different from those published by Zhu et al [16]. There are quantitative differences in the inhibition of NF-κB activation by optineurin. The major difference is that in our study we did not see inhibition of basal NF-κB activity by overexpressed optineurin in HeLa cells. This observation has been reported earlier by us [18]. Zhu et al have used HaLa S3 cells and this difference could be due to differences in the cell lines used. However, we and Zhu et al have shown that knockdown of optineurin increases basal NF-κB activity in various cells though the mechanism by which this occurs was not known. We have now shown that this increase in basal activity is possibly due to accumulation of ubiquitinated RIP in optineurin knockdown cells.

Though mutations in optineurin have been associated with glaucoma and more recently with amyotrophic lateral sclerosis [28], [29], [37], [38], the functional defects caused by these mutations are not completely understood. The H486R mutation is associated with certain types of glaucomas [37], [38]. Our observations with H486R mutant show that this mutant is defective in its interaction with CYLD. In accordance with the role of optineurin in CYLD mediated inhibition of NF-κB activation, this mutant is unable to abrogate NF-κB activation. As the H486R mutation is located within the UBD, this mutant showed some loss in ubiquitin binding and in binding to ubiquitinated RIP. Thus the inability of the H486R mutant to inhibit NF-κB activation is mainly due to its altered interaction with CYLD although reduced binding to ubiquitin may also contribute to some extent. This assumption is strengthened by our results which show that overexpressed CYLD fails to deubiquitinate RIP and inhibit TNFα-induced NF-κB activation in presence of H486R mutant.

How does the H486R mutant cause glaucoma? Glaucoma is a neurodegenerative disease in which loss of vision occurs due to death of retinal ganglion cells in the optic nerve head. Several mechanisms are implicated in retinal ganglion cell death in glaucoma including direct effect on retinal ganglion cells, activation of glial cells to produce cytotoxic molecules like TNFα, alteration in trabecular meshwork, autoimmunity etc [39], [40]. Since overexpression of H486R mutant does not induce retinal ganglion cell death unlike the E50K mutant [41], it is plausible that indirect effects on other cells might contribute to H486R induced glaucomatous neurodegeneration. NF-κB is activated in glaucomatous trabecular meshwork cells and is also associated with autoimmune responses [42]–[44]. Our results showing defective NF-κB regulation by H486R mutant optineurin thus provide a basis for exploring its role in indirect mechanisms of glaucomatous degeneration.

In conclusion, our studies show that optineurin acts as an adaptor protein to bring together CYLD and its substrate RIP. The interaction of CYLD with optineurin is essential for negative regulation of TNFα-induced NF-κB activation. A defect in this interaction, as observed in the glaucoma associated H486R mutant results in deregulation of NF-κB. These findings may have relevance to the pathogenesis of glaucoma, directly or indirectly, as deregulation of NF-κB activity has been implicated in glaucomas.

## Materials and Methods

### Cell culture and transfections

Cell lines were maintained at 37°C in a CO_2_ incubator in Dulbecco's modified Eagle's medium supplemented with 10% foetal bovine serum. Transfections were carried out using Lipofectamine Plus reagent (Invitrogen, San Diego, CA, USA) according to the manufacturer's instructions. All the plasmids for transfection were prepared by using Qiagen columns (Hilden, Germany). Human TNFα (Sigma, St. Louis, MO, USA or Calbiochem) was added wherever indicated at a final concentration of 10–20 ng/ml.

### Expression vectors and antibodies

Human optineurin expression plasmid and its mutants cloned in various vectors have been described by us previously [21], [41]. Deletion constructs of optineurin were generated by PCR. HA-tagged CYLD and GFP-CYLD were generated by cloning CYLD c-DNA obtained in yeast-two hybrid screen in pCDNA3.1-HA or pEGFP-C2, respectively. The catalytically inactive H871N mutant of CYLD was generated by site directed mutagenesis. Rabbit polyclonal optineurin antibody (ab23666) was from Abcam, mouse monoclonal RIP antibody (Cat no 610459) was from BD Biosciences and mouse monoclonal Lys63-sepcific anti-ubiquitin was from Miilipore (Cat no 05-1313). IκB-α (sc-371), actin and Cdk2 antibodies were from Santa Cruz Biotechnology (Santa Cruz, CA, USA).

### Generation of adenoviral vectors

The adenoviral shRNA expression vectors (Ad-shOPTN1 & 2) for targeting human optineurin, were generated by using pAdEasy system and have been described by us [18], [21]. The shRNAs generated by these adenoviruses target two different regions of optineurin mRNA [18]. As a control, an adenovirus expressing shRNA of unrelated sequence of the same length was used. Adenoviral vectors for expressing optineurin and its mutants with HA tag were prepared as described [21], [45]. These adenoviruses also express GFP for monitoring of infection. For overexpression experiments, an adenovirus expressing GFP was used as a control.

### Yeast two-hybrid assay

Yeast two-hybrid assay was performed as described previously [46]. Briefly, yeast strain PJ69-4A was co-transformed with required plasmids by lithium-acetate method. Optineurin, its mutants and deletion constructs were cloned in pGBKT7 (GAL4 DNA binding domain) while CYLD was cloned in pACTII (GAL4 activation domain) (Clonetech). The transformants were selected by growth in minimal media (Trp^−^, Leu^−^). Yeast colonies obtained on Trp^−^, Leu^−^ plates were patched onto selection plants (Trp^−^, Leu^−^ Ade^−^) and X-Gal (Trp^−^, Leu^−^, X-Gal^+^) plates to assay activation of reporter genes and hence interaction. Growth on Ade^−^ plate or colour on X-Gal plate indicated interaction.

### Immunoprecipitation and GST pull down

Immunoprecipitations were carried out essentially as described [21], [46]. Briefly, cells were lysed in lysis buffer (25 mM Tris pH 7.4, 1% Triton X-100, 150 mM NaCl, 0.1% BSA, 1 mM PMSF, 10 mM N-ethylmaleimide, 2 mM sodium vanadate, 25 mM NaF and protease inhibitor cocktail (Roche)) and immunoprecipitations were carried out with 2 µg of appropriate antibodies overnight at 4°C (2.5 h at 4°C for RIP). The immunoprecipitated proteins were washed 3 times with lysis buffer, eluted by boiling in SDS sample buffer and resolved in 8–10% SDS-PAGE. The proteins were transferred to nitrocellulose membrane for western blot analysis as described [18].

For GST pull down assays, GST and GST-fusion proteins were expressed in *E. coli* and were conjugated to sepharose beads as described [21], [47]. These beads were incubated for 6–8 hours with lysates of HeLa cells transiently transfected with indicated plasmids. Bound proteins were eluted by boiling in SDS sample buffer and subjected to immunoblotting.

### Reporter assays

NF-κB reporter assays were performed as described previously by using a reporter plasmid NF-κB-Luc containing five tandem NF-κB binding sites upstream of a luciferase gene [18]. HeLa cells grown in 24 well dishes were transfected with 25 ng of the NF-κB-luc plasmid, 50 ng of β-galactosidase expression plasmid along with the required amount of other plasmids [18]. Relative luciferase activities were calculated after normalizing with β-galactosidase enzyme activities. TNFα was used at a concentration of 20 ng/ml.

### Statistical analysis

Graphs represent average ± SD values. Statistical differences were calculated using student's T-test. When significant differences were observed, P values for pair wise comparisons were calculated by using two-tailed T-test. P value less than 0.05 was considered significant.
